# Characterization of Oxidative Guanine Damage and Repair in Mammalian Telomeres

**DOI:** 10.1371/journal.pgen.1000951

**Published:** 2010-05-13

**Authors:** Zhilong Wang, David B. Rhee, Jian Lu, Christina T. Bohr, Fang Zhou, Haritha Vallabhaneni, Nadja C. de Souza-Pinto, Yie Liu

**Affiliations:** 1 Laboratory of Molecular Gerontology, National Institute on Aging, National Institutes of Health, Baltimore, Maryland, United States of America; 2 University of Tennessee–Oak Ridge National Laboratory Graduate School of Genome Science and Technology, Knoxville, Tennessee, United States of America; University of Washington, United States of America

## Abstract

8-oxo-7,8-dihydroguanine (8-oxoG) and 2,6-diamino-4-hydroxy-5-formamidopyrimidine (FapyG) are among the most common oxidative DNA lesions and are substrates for 8-oxoguanine DNA glycosylase (OGG1)–initiated DNA base excision repair (BER). Mammalian telomeres consist of triple guanine repeats and are subject to oxidative guanine damage. Here, we investigated the impact of oxidative guanine damage and its repair by OGG1 on telomere integrity in mice. The mouse cells were analyzed for telomere integrity by telomere quantitative fluorescence in situ hybridization (telomere–FISH), by chromosome orientation–FISH (CO–FISH), and by indirect immunofluorescence in combination with telomere–FISH and for oxidative base lesions by Fpg-incision/Southern blot assay. In comparison to the wild type, telomere lengthening was observed in *Ogg1* null (*Ogg1^−/−^*) mouse tissues and primary embryonic fibroblasts (MEFs) cultivated in hypoxia condition (3% oxygen), whereas telomere shortening was detected in *Ogg1^−/−^* mouse hematopoietic cells and primary MEFs cultivated in normoxia condition (20% oxygen) or in the presence of an oxidant. In addition, telomere length abnormalities were accompanied by altered telomere sister chromatid exchanges, increased telomere single- and double-strand breaks, and preferential telomere lagging- or G-strand losses in *Ogg1^−/−^* mouse cells. Oxidative guanine lesions were increased in telomeres in *Ogg1^−/−^* mice with aging and primary MEFs cultivated in 20% oxygen. Furthermore, oxidative guanine lesions persisted at high level in *Ogg1^−/−^* MEFs after acute exposure to hydrogen peroxide, while they rapidly returned to basal level in wild-type MEFs. These findings indicate that oxidative guanine damage can arise in telomeres where it affects length homeostasis, recombination, DNA replication, and DNA breakage repair. Our studies demonstrate that BER pathway is required in repairing oxidative guanine damage in telomeres and maintaining telomere integrity in mammals.

## Author Summary

It has been proposed that oxidative DNA damage compromises telomere integrity. However, neither the types nor the consequences of oxidative DNA damage in telomeres have been fully explored. Oxidative base lesions, especially 8-oxoG and FapyG are among the most common oxidative DNA damage. These guanine lesions are excised by a DNA glycosylase, OGG1, and subsequently replaced by normal guanines via BER pathway. Telomeric DNA is rich in guanine, and guanine base is prone to be oxidized to 8-oxoG and FapyG. To study if 8-oxoG and FapyG accumulate in telomeres and affect telomere integrity in mammals, we utilized a mouse model that was genetically deleted for the *Ogg1* gene (it is thus defective in 8-oxoG and FapyG repair). We found that oxidative guanine lesions accumulated in telomeres in *Ogg1* deficient mouse cells, which was associated with multiple telomere defects. These findings demonstrate that oxidative guanine damage can perturb telomere integrity and that BER pathway actively participates in oxidative base repair in telomeres in mammals. Our studies will be beneficial for uncovering the role of oxidative DNA damage and BER in mammalian telomeres.

## Introduction

Telomeres are chromosome end nucleoprotein structures that are composed of telomere associated proteins and TTAGGG repeats in mammals [1]. Telomeres cap chromosome ends and prevent them from being recognized as broken DNA. Dysfunctional telomeres, emanating from loss of telomere repeats and/or loss of protection by telomere-associated proteins, are recognized by many DNA damage response proteins, including γH2AX, 53BP1, and ATM that form telomere dysfunction-induced foci (TIF) and induce cellular senescence or apoptosis [2]–[3].

Telomere length homoeostasis is maintained through interplay among telomerase extension, telomere recombination, telomere replication, and telomere capping [1]. Telomerase, a ribonucleoprotein complex, replenishes replication dependent-telomere repeat loss and is essential in telomere length maintenance [1]. Telomere associated proteins also play a key role in telomere length regulation and capping. Mammalian telomeres are coated by a telomere protein complex, referred as shelterin. Shelterin includes telomere binding proteins TRF1, TRF2, and POT1 [3] that negatively control telomere length *in cis* by limiting the access of telomerase to the ends of individual telomeres [4]–[7]. Reduced telomere-bound TRF1 promotes telomere lengthening in human cells [4]–[5], but telomeres that are severely or completely stripped off the protective telomere protein complex result in telomere uncapping and evoke ATM or ATR dependent DNA damage response, nucleolytic degradation and undesirable recombination [8]–[15]. Efficient telomere replication also requires the telomere associated proteins, e.g. TRF1 and WRN [16]–[19].

Oxidative stress has been proposed to be a major cause of telomere shortening in cultured cells [20]. For instance, normoxia, hyperoxia (40% oxygen), and mitochondrial dysfunction-induced reactive oxygen species (ROS) accelerate telomere shortening and severely reduce proliferative lifespan of human somatic cells *in vitro*; while these phenotypes are delayed when cells are grown in hypoxia or in the presence of antioxidants [20]. Interestingly, human cells with long telomeres show increased sensitivity to hydrogen peroxide, but not to etoposide and bleomycin, supporting the notion that telomeres are particularly vulnerable to oxidative damage [21]. These studies suggest that oxidative stress causes telomere shortening or damage; however it is unclear which types of oxidative DNA damage arise in telomeres and how they compromise telomere length and integrity. Previous studies have demonstrated that oxidative stress causes single strand breaks (SSBs) in telomeric DNA [22]. Thus, telomere shortening could arise from SSBs. Oxidative stress has also been shown to induce oxidative base damage in telomeric oligonucleotides *in vitro* [23]–[25], and 8-oxoG at double-stranded telomeric nucleotides attenuates binding by TRF1 and TRF2 [26]. It is unclear if oxidative base damage has any impact on telomere length and integrity in mammalian cells.

Oxidative DNA damage, resulting from ROS, increases with age and can accumulate as a variety of oxidative modifications in purines and pyrimidines [27]–[28]. Oxidized bases may lead to mutagenesis, block DNA replication, or alter the affinity of DNA binding proteins, which can, in turn, attenuate cell viability or promote tumorigenesis [29]–[31]. BER is the primary DNA repair pathway for the repair of non-bulky damaged bases, and the initial step in BER is base removal by a DNA glycosylase. Several DNA glycosylases with distinct, but overlapping substrate specificities have been characterized, and OGG1 primarily excises 8-oxoG and FapyG paired with cytosine in duplex DNA [30]–[31]. OGG1 is well conserved from bacterial to mammals, implying its significant functional importance in maintaining genome integrity [27]–[28]. If 8-oxoG is unrepaired, it becomes highly mutagenic, because it can pair with adenine and lead to GC to TA transversions after two rounds of replication [32]–[34]. The removal of adenine opposite 8-oxoG is via the adenine-specific mismatch DNA glycosylase, MYH [35]. Mice lacking these repair genes exhibit an increased spontaneous mutation rate and a marked increase in tumor predisposition [35]–[38]. In addition, *Ogg1* and *Myh* deficient murine cells are sensitive to oxidative stress [39]–[41]. These studies are consistent with the idea that oxidative base lesions contribute to genome instability, neoplastic transformation, and cell death.

*Ogg1* deficiency causes an increase in 8-oxoG and FapyG lesions in the mouse genome [29], [36], [42]. This genetic model therefore allows us to study whether these unique oxidative guanine lesions can affect telomere integrity. Here, we present evidence that deletion of the mouse *Ogg1* gene attenuates telomere integrity via multiple ways. Thus, interfering with telomere integrity may be one of the mechanism(s) by which oxidative base damage leads to genome instability.

## Methods

### Mice and primary mouse cells

The generation of *Ogg1* null mice was described elsewhere [36]. *Ogg1^−/−^* mice were further backcrossed into C57BL/6 background. Wild type and *Ogg1^−/−^* mice were derived from heterozygous (*Ogg1^+/−^*) breeders. Primary MEFs were isolated from 13.5 day embryos of *Ogg1^+/−^* female bred with *Ogg1^+/−^* male and cultured in Dulbecco's Modified Eagle Medium containing 10% fetal bovine serum. Splenocytes were prepared from mouse spleens, cultured in RPMI 1640 with 10% FBS and 0.1% 2-mercaptoethanol, and stimulated with 50 µg/ml *Escherichia coli* LPS serotype O111:B4 (Sigma-Aldrich) and 50 ng/ml mouse IL-4 (R&D Systems). Bone marrow cells were flushed from femurs and tibias and cultured with Iscove's modified Dulbecco's medium (IBCO-BRL) supplemented with 20% fetal calf serum (Hyclone) in the presence of interleukin 6 (200 U/mL; Peprotech) and stem cell factor (100 ng/mL; Peprotech). To decrease or enhance oxidative stress, mouse cells were cultured in 3% oxygen (SANYO O_2_/CO_2_ incubator, MCO-18M) or 20% oxygen or in the presence of paraquat. All animal experiments were carried out according to the “Guide for the Care and Use of Laboratory Animals” (National Academy Press, USA, 1996), and were approved by the Institutional Animal Care and Use Committee of National Institute on Aging.

### Telomere quantitative fluorescence in situ hybridization

The telomere fluorescence in cell populations of spleen, bone marrow, and primary MEFs was measured by Flow cytometry and FISH (Flow-FISH) according to previously published protocol [43]. A telomere specific FITC conjugated (CCCTAA)_3_ PNA probe (0.3 µg/ml, Panagene) was used.

Quantitative FISH (Q-FISH) was performed as previously described [44]–[46]. Metaphase spreads were prepared from freshly isolated or subcultured mouse bone marrow cells, activated splenocytes, and primary MEFs. Briefly, mice were injected with 100 µl of 0.5% colchicine intraperitoneally for approximately 30 minutes before being sacrificed. Bone marrow cells were then collected by flushing 1ml of PBS through femurs and tibias. Cultured mouse cells were incubated with 0.1 µg/ml colcemid for 2–6 hr at 37°C to allow mitotic cells to accumulate. Metaphase spreads were obtained by incubating colchicine- or colcemid- treated mouse cells in 0.075 M KCl for 15 minutes in 37°C, followed by fixing cells in ice-cold 3∶1 methanol and glacial acetic acid and dropping the fixed cells onto slides. Metaphase spreads were hybridized with Cy3-labeled (CCCTAA)_3_ (0.3 µg/ml, Panagene), washed, and then counterstained with 4,6 diamidino-2-phenylindole (DAPI). For the detection of telomere signal intensity in G and C strands, metaphase spreads were initially hybridized with FITC-labeled (CCCTAA)_3_ PNA probes (0.3 µg/ml, Panagene). The free-(CCCTAA)_3_ probe were washed off the slides, and then hybridized with TRAMA-labeled (TTAGGG)_3_ (0.3 µg/ml, Panagene). Images were captured using Cytovision software (Applied Imaging) on a fluorescence microscope (Axio2; Carl Zeiss); followed by quantification of telomere fluorescence signals using the TFL-Telo software (a kind gift from Dr. Peter Lansdorp). For histograms and box-plots, data from different mice of each genotype were scored and R statistical package (http://www.r-project.org/) along with R.utils package and Biobase package (http://www.bioconductor.org/) were used. The frequencies of telomeres within a given range of telomere signal intensities were plotted against the telomere signal intensity using arbitrary units. Metaphases from different mice of each genotype were scored for chromosomal and telomeric abnormalities as previously described [45]–[46].

### Telomerase activity

Telomerase activity was measured by Biomax Telomerase Detection Kit (Biomax) according to the manufacturer's recommendations. Briefly, mouse cell extracts were added to a pre-mix for quantitative telomerase activity in a real-time PCR reaction. MyiQ Single-Color Real-Time PCR Detection System (Bio-Rad) was used to perform the reactions, where each sample was done in triplicates and performed according to the manufacturer's instructions. HeLa cell extracts were used as positive control. *Tert* knockout mouse cell extracts and RNase-treated HeLa cell extracts were used as negative controls. Relative telomerase activity was expressed as log of C_T_ value.

### Measurement of telomere sister chromatid exchanges (T–SCEs)

CO-FISH was used to measure T-SCEs and telomere lagging or leading strand loss [16], [47]. Briefly, mice were injected with 3∶1 ratio of BrdU/BrdC (Sigma) at a final concentration of 1×10^−5^ M intraperitoneally for approximately 20 hours, and subsequently with 100 µl of 0.5% colchicine for approximately 30 minutes before being sacrificed. Bone marrow cells were then collected by flushing 1ml of PBS through femurs and tibias. MEFs were cultured in medium containing a 3∶1 ratio of BrdU/BrdC (Sigma) at a final concentration of 1×10^−5^ M for 24 hours, and colcemid (0.1 µg/ml) was added 4 hours before harvest. Metaphase spreads were prepared from mouse bone marrow cells or MEFs, stained with Hoechst 33258, exposed to UV light, and digested with exonuclease III to remove newly synthesized DNA strands. Hybridization and wash conditions were identical to those described for Q-FISH. FITC-labeled (CCCTAA)_3_ and TRAMA-labeled (TTAGGG)_3_ PNA probes were used for the detection of lagging and leading strand, respectively. A chromosome with more than two telomeric DNA signals by both FITC-labeled (CCCTAA)_3_ and TRAMA-labeled (TTAGGG)_3_ PNA probes was scored as T-SCE positive. A chromosome with loss of one or two telomeric DNA signals by either FITC-labeled (CCCTAA)_3_ or TRAMA-labeled (TTAGGG)_3_ PNA probes was scored for telomere lagging or leading strand loss.

### Indirect immunofluorescence and telomere FISH (TEL–FISH)

TEL-FISH was performed as described previously [46] with minor modifications. Briefly, cells were fixed in 1∶1 methanol∶acetone (Sigma) at −20°C for 10 minutes, permeabilized with 0.5% NP-40, and blocked in 1% Bovine serum albumin (BSA) (IgG-free, Sigma). Cells were first immunostained with a rabbit anti-γH2AX antibody (16193, Upstate Biotechnology), a rabbit anti-53BP1 antibody (BN 100–304, Novus Biologicals), or a mouse anti-XRCC1 antibody (X0629, Sigma) overnight at 4°C followed by Alexa 488-labeled secondary antibody (1∶500; Molecular Probes) for one hour at 37°C. Slides were washed with PBS for 15 minutes, fixed in 2% paraformaldehyde at room temperature for 10 minutes, dehydrated through ethanol series, and air-dried briefly. Slides were then hybridized to a TRAMA-labeled (CCCTAA)_3_ PNA probe (Panagene), then counterstained with DAPI. Z-stack images were captured and deconvoluted using Axiovision 4.6.3 software on a fluorescence microscope (Axiovert 200M; Carl Zeiss).

### Detection of oxidative base lesions in telomeres

Identification of oxidative base lesions in telomeres was performed as previously described [48] with modifications. In brief, DNA was isolated from mouse liver or primary MEFs by salting out. 4 µg of DNA was treated with *HinfI* and *RsaI* restriction enzyme at 37°C overnight. The reaction was heated at 65°C for 15 minutes and then divided into two equal portions; one was treated with 8 units of *E. coli* formamidopyrimidine-DNA glycosylase (Fpg) (New England Biolabs) and another was treated with a mock buffer at 37°C for 30 minutes. Fpg was inactivated by heating at 60°C for 15 minutes. Genomic single-stranded DNA fragments were separated on 1% alkaline agarose gel according to their sizes, treated with UV light, then transferred to a nylon membrane. Single-stranded telomere DNA fragments were detected by ^32^P-labeled (CCCTAA)_4_ probe and visualized by autoradiography. ImageQuant software was applied in quantifying DNA cleavage in mock and Fpg-treated samples. A grid object was created as a single column with multiple rows and was placed over the lane corresponding to the molecular size markers. The density measurement was conducted in each row in which each marker was recorded. The mean length (ML) was calculated as a center of mass and expressed in kb: ML = Σ (MWi × ODi)/Σ (ODi), in which MWi is the length of the telomeric DNA at each row and ODi is the densitometer output at each row. The frequencies of Fpg-sensitive lesions in a sample were calculated based on ML values in Fpg- and mock-treated samples: lesions = (ML untreated/ML treated)−1, in which ML is expressed in kb. Fold-changes in each sample were further normalized with respect to the number of Fpg-sensitive lesions in a control.

## Results

### *Ogg1*-deficient mouse tissues and primary MEFs under low oxygen tension display telomere lengthening

**Figure 1:**
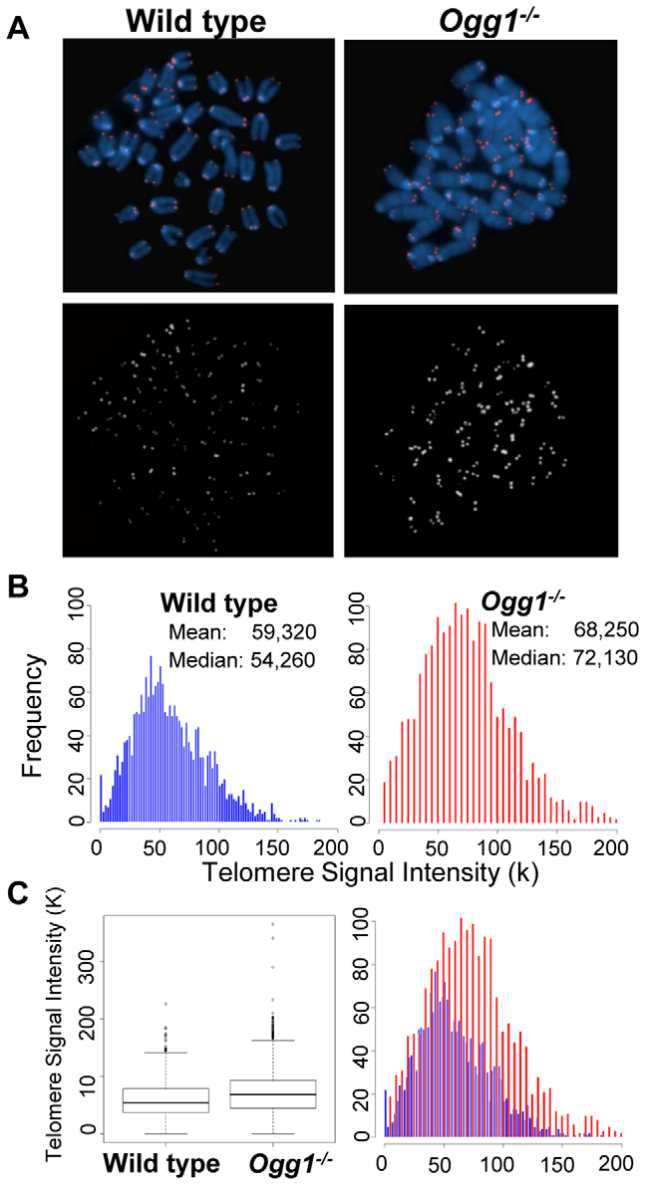
Telomere length in hematopoietic cells freshly isolated from wild-type and *Ogg1^−/−^* mice. Q-FISH analysis of metaphase spreads of the mouse bone marrow cells from wild type and *Ogg1^−/−^* mice (n = 6). (A) Representative metaphase spreads of wild type and *Ogg1^−/−^* mouse bone marrow cells showing DAPI staining (blue, upper panel) and telomere fluorescence signals (red, upper panel; white, lower panel). Representative quantitative measurement and dynamic range of telomeric DNA signal intensity at individual chromosome ends in a wild type and an *Ogg1^−/−^* mouse are shown as histogram (B) and box-plot (C). An increase in telomere signal intensity was repeatedly observed in *Ogg1^−/−^* mice, compared to age-matched wild type mice (1.5-, 1.3-, 1.2-fold increase in three pairs of mice, respectively).

**Figure 2:**
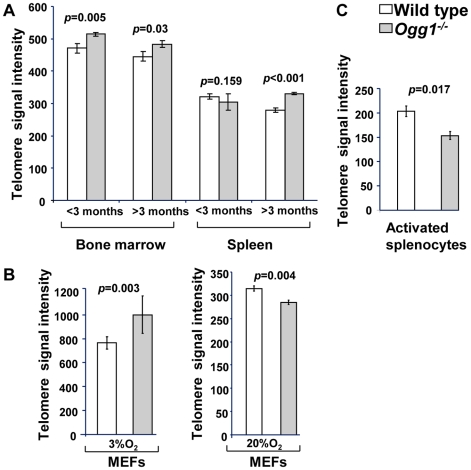
Average telomere length in wild-type and *Ogg1^−/−^* mouse tissues and primary MEFs. Flow-FISH analysis of telomere signal intensity in freshly isolated bone marrow and spleen from wild type and *Ogg1^−/−^* mice (n = 15) at different age (A), wild type and *Ogg1^−/−^* primary MEFs cultivated in 3% and 20% oxygen for 6 passages (B), and wild type and *Ogg1^−/−^* splenocytes cultivated in 20% oxygen for 3 days (C). Telomere signal intensity was altered in *Ogg1^−/−^* mouse tissues and cells, compared to the wild type control.

**Figure 3:**
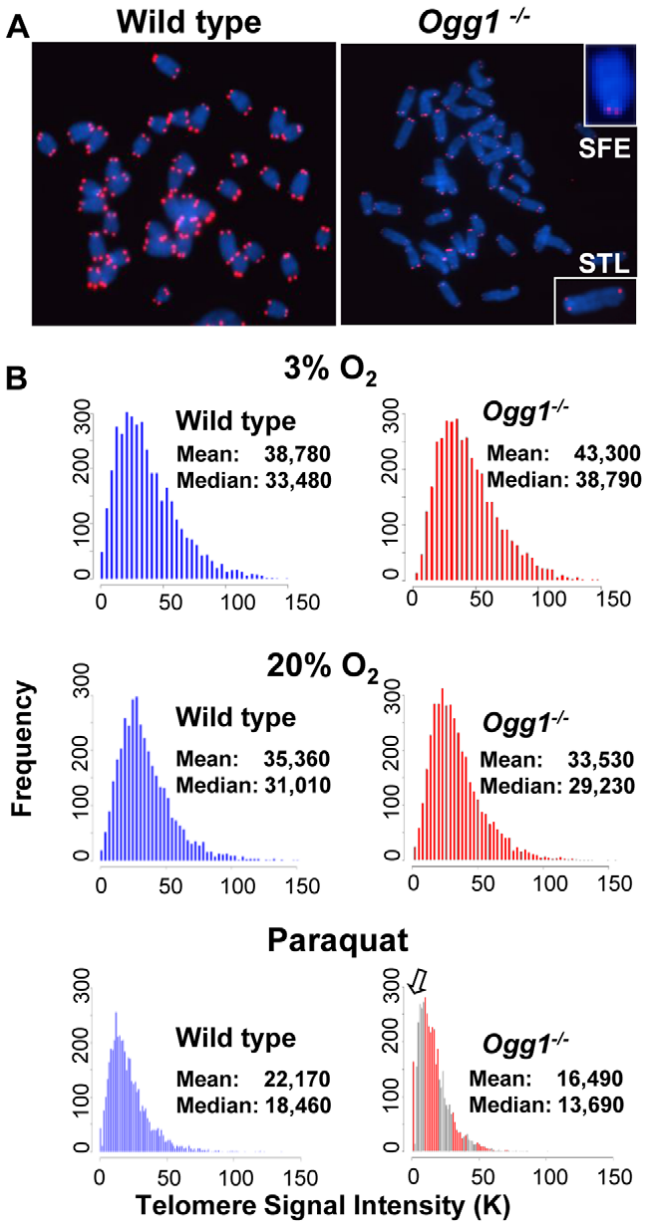
Telomere length in primary MEFs cultivated in low or high oxidative environment. Q-FISH analysis of metaphase spreads of wild type and *Ogg1^−/−^* primary MEFs cultivated in 3% O_2_, 20% O_2_, or 3% O_2_ in the presence of 0.5 µM paraquat for 6 passages. (A) Representative metaphase spreads of wild type and *Ogg1^−/−^* primary MEFs cultivated in 20% O_2_. Loss of telomere signals at chromosomes or chromatids were more frequently observed in *Ogg1^−/−^* MEFs (see enlarged chromosomes in boxes). (B) Representative histogram of dynamic range of telomeric DNA signal intensity at individual chromosome ends in a wild type and an *Ogg1^−/−^* primary MEF line. In comparison to wild type MEFs, slight increase in telomere signal intensity was repeatedly detected in *Ogg1^−/−^* MEFs in 3% O_2_ (approximately 1.1- fold increase in three pairs of MEF lines); and decreased telomere signal intensity and increased SFEs (see arrow) were repeatedly observed in *Ogg1^−/−^* MEFs cultivated with 20% O_2_ (2-, 1.7-, and 1.4-fold decrease in three pairs of MEF lines, respectively) and 0.5 µM paraquat (1.8-, 1.3-, and 1.2- fold decrease in three pairs of MEF lines, respectively).

Ablation of OGG1 function in *S. cerevisiae* can cause telomere elongation [49]–[50]. Since OGG1 is conserved from *S. cerevisiae* to mice, we investigated the impact of *Ogg1* deficiency on telomere length in mice. Mouse bone marrow cells were freshly isolated from 1–3 month old mice and analyzed by Q-FISH. Compared to the wild type, *Ogg1^−/−^* mouse bone marrow displayed higher mean and median telomere signal intensities (Figure 1). Similar results were obtained from bone marrow cells from 12 month old mice (Figure S1). This observation was further confirmed by Flow-FISH, showing that telomere signal intensity was moderately increased in *Ogg1^−/−^* mouse bone marrow cells from young and old animals (Figure 2A). Freshly isolated splenocytes from >3 month old *Ogg1^−/−^* mice also displayed higher telomere signal intensity than those of age-matched wild type mice (Figure 2A). Additionally, we examined telomere length in wild type and *Ogg1^−/−^* primary MEFs cultivated in 3% O_2_ that mimics the *in vivo* oxygen level in mice. Telomere signal intensity was moderately increased in *Ogg1^−/−^* primary MEFs as shown by Flow-FISH (Figure 2B) and, to a lesser extent, by Q-FISH (Figure 3).

Next, we examined if deletion of *Ogg1* could affect telomere capping *in vivo*. Freshly isolated mouse bone marrow cells and primary MEFs cultivated in 3% O_2_ were examined for the frequencies of chromosome end-to-end fusions and telomere signal free ends (SFEs). *Ogg1^−/−^* mouse cells did not show any chromosome end-to-end fusions. The incidence of SFEs was low and not significantly different between wild type and *Ogg1^−/−^* mouse cells. Furthermore, *Ogg1^−/−^* mouse cells did not display spontaneous chromosomal abnormalities, e.g. chromosome breaks or fragments (Table 1). These data suggest that ablation of OGG1 function does not lead to telomere uncapping and chromosomal instability, but moderate telomere lengthening in mouse tissues and primary cells that are subjected to low levels of oxidative stress.

**Table 1:** Frequencies of chromosomal and telomeric abnormalities in wild-type and *Ogg1^−/−^* bone marrow cells and primary MEFs.

Cell types	Fragments & breaks	End-to-end fusion	SFEs
***Bone Marrow***			
Wild type	0% (0/1919	0% (0/1919)	0.4% (7/1919)
*Ogg1^−/−^*	0% (0/1980)	0% (0/1980)	0.7% (14/1980)
***Primary MEFs (*** **3% O_2_** ***)***			
Wild type	0% (0/1087)	0% (0/1087)	3.2% (35/1087)
*Ogg1^−/−^*	0% (0/1140)	0% (0/1140)	4.1% (47/1140)
***Primary MEFs (20% O2)***			
Wild type	0.1% (1/846)	0% (0/846)	4.5% (38/846)
*Ogg1^−/−^*	0.7% (7/946)	0.11% (1/946)	8.4% (79/946)*
***Primary MEFs (0.5*** ** µ** ***M paraquat)***			
Wild type	0.1% (1/993)	0.1% (1/993)	4.1% (41/993)
*Ogg1^−/−^*	0% (0/983)	0.2% (2/983)	18.6% (183/983)*

Abnormal events/total chromosomes were shown in parentheses. SFEs: telomere signal free ends. The data were obtained from metaphase spreads and Q-FISH analysis of different mice (n = 6) or MEF lineages (n = 6) of each genotype. * *P*-value between wild type and *Ogg1^−/−^* yielded a statistical difference in indicated categories.

### High oxidative stress increases telomere attrition in *Ogg1*-deficient mouse cells

To determine if high oxidative stress has the same or different impact on telomere length, primary wild type and *Ogg1^−/−^* MEFs were cultivated in 20% O_2_ or in the presence of 0.5 µM of paraquat (an oxidant). After six passages, they were evaluated for telomere length by Q-FISH and Flow-FISH. Surprisingly, under these conditions *Ogg1^−/−^* MEFs showed reduced telomere signal intensity, in comparison to wild type MEFs (Figure 2B and Figure 3). In addition to overall reduction in telomere signal intensity, *Ogg1^−/−^* MEFs had increased number of chromosomes and chromatids without detectable telomere signals (referred to SFEs and sister telomere loss, STL, respectively) (Figure 3 and Table 1). Notably, wild type MEFs also showed reduced telomere signal intensity after prolonged exposure to 20% O_2_ and 0.5 µM paraquat, in comparison to 3% O_2_. Nevertheless, they had less degree of telomere loss than *Ogg1^−/−^* MEFs (Figure 3). Similarly, subcultured *Ogg1^−/−^* mouse splenocytes and bone marrow cells showed reduced telomere signal intensity than wild type splenocytes, after being exposed to 20% O_2_ for the period of three days or to 200 µM paraquat for 16 hours, respectively (Figure 2C, Figure S2, and Figure S3). Collectively, these results suggest that high oxidative stress increases telomere attrition in *Ogg1^−/−^* mouse cells.

### Telomere recombination is altered in *Ogg1*-deficient mice

**Figure 4:**
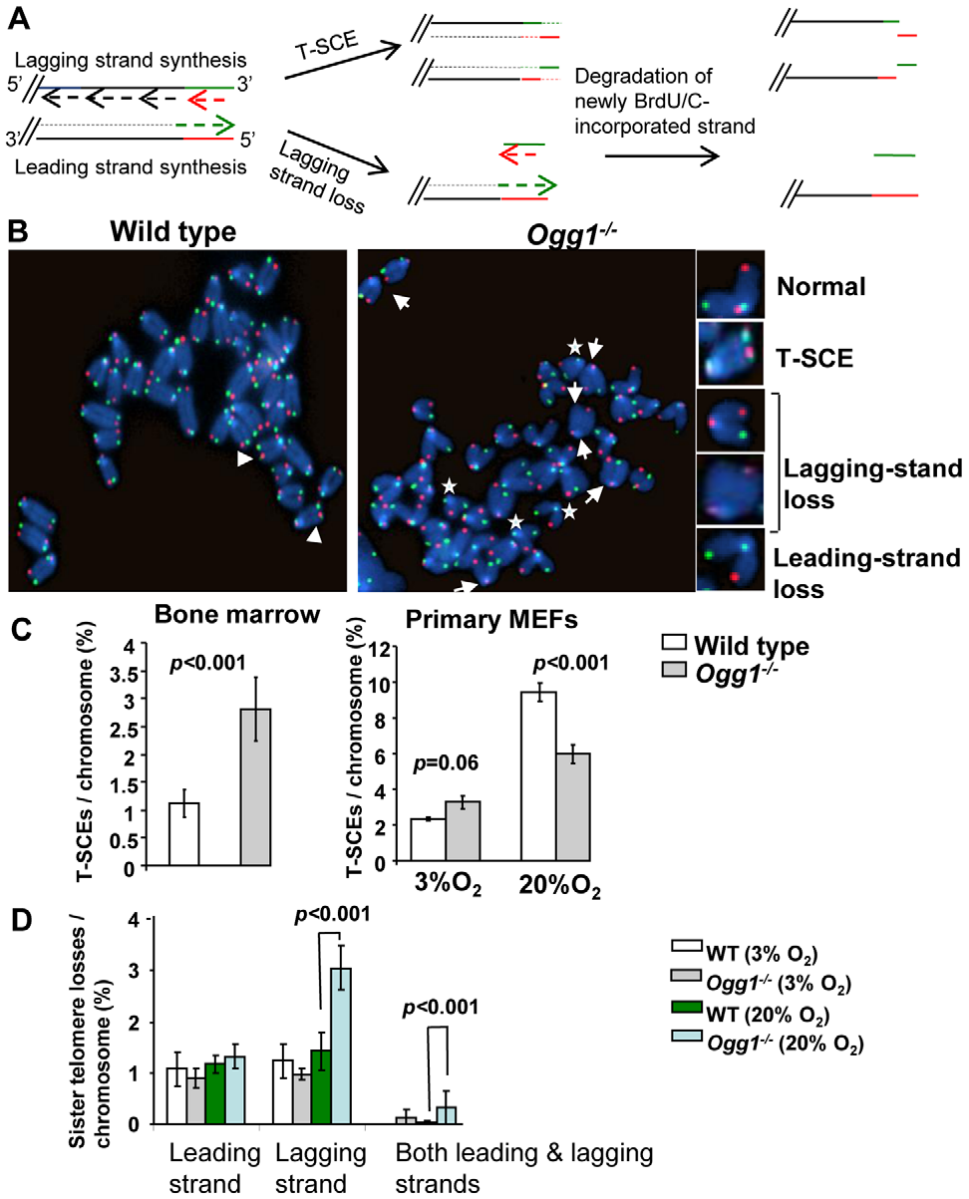
T-SCEs and telomere lagging or leading strand loss in wild-type and *Ogg1^−/−^* mouse cells. (A) A schematic presentation of CO-FISH. In brief, newly synthesized strands are removed, leaving parental strands to be detected by TRAMA-labeled (TTAGGG)_3_ PNA probe (red color) and FITC-labeled telomere (CCCTAA)_3 PNA_ probe (green color). In an event of T-SCE, an end shows telomere signals in both green and red. In the event of collapse in telomere lagging strand synthesis, an end displays loss or reduction in telomere signal intensity in lagging strand. (B) Representative metaphase spreads of wild type and *Ogg1^−/−^* primary MEFs showing DAPI staining (blue), leading strand telomere fluorescence signals (red) and lagging strand telomere fluorescence signals (green). Arrow head: T-SCEs. Arrow: telomere lagging strand loss. *: telomere leading strand loss. (C) The frequencies of T-SCEs in freshly isolated wild type and *Ogg1^−/−^* mouse bone marrow cells (left panel) and primary MEFs cultured in 3% O_2_ or 20% O_2_ (right panel). (D) The frequencies of telomeric loss in either telomere lagging or leading strand or both strands within a chromosome in wild type and *Ogg1^−/−^* primary MEFs cultured in 3% O_2_ or 20% O_2_. When cultivated in 20% O_2_, *Ogg1^−/−^* MEFs showed significant increase in lagging strand loss, compared to wild type MEFs.

8-oxoG in telomeric DNA attenuates binding by telomere binding proteins [26], which may consequently evoke undesirable telomere recombination [14]–[15]. On the other hand, *Ogg1* deficiency may hamper telomere recombination [51]. We thus examined the frequencies of telomere sister chromatid exchange (T-SCE) in wild type and *Ogg1^−/−^* mouse cells using CO-FISH (Figure 4A and 4B, and [47]). Freshly isolated *Ogg1^−/−^* bone marrow cells showed moderately increased T-SCEs (1.12±0.25% and 2.82±0.57% T-SCEs/chromosome in wild type and *Ogg1^−/−^*, respectively, *p*<0.001) (Figure 4C). In 3% O_2_ primary *Ogg1^−/−^* MEFs displayed slight yet insignificant increase in T-SCE events (2.35±0.08% and 3.28±0.35% T-SCEs/chromosome in wild type and *Ogg1^−/−^* respectively, *p* = 0.06) (Figure 4C). In 20% O_2_ primary *Ogg1^−/−^* MEFs, however, had fewer T-SCEs than the wild type (5.98±0.52% and 9.43±0.51% T-SCEs/chromosome in *Ogg1^−/−^* and wild type, respectively, *p*<0.001) (Figure 4C). These results suggest that deletion of *Ogg1* may induce or inhibit telomere recombination, possibly depending on the level of oxidative stress.

### Telomerase activity is not altered in *Ogg1*-deficient mice

Telomerase plays a key role in telomere elongation [1]. Telomere lengthening in *ogg1*-deleted *S. cerevisiae* is dependent on telomerase [50]. We therefore examined if telomerase activity was altered in *Ogg1^−/−^* mice. No detectable differences in telomerase activity were observed between wild type and *Ogg1^−/−^* mouse bone marrow cells by qT-PCR TRAP assay (Figure S4). Telomere lengthening is therefore unlikely through enhanced telomerase activity in *Ogg1^−/−^* mice; however, we cannot exclude the possibility that there is an increased accessibility of telomerase to telomeres in *Ogg1^−/−^* mice.

### High oxidative stress enhances telomeric DNA strand breaks in *Ogg1*-deficient primary MEFs

Previous studies suggest that oxidative stress-induced SSBs could result in telomere shortening [22]. In addition, oxidative base damage in the vicinity of DNA breaks can impose hindrance for resolving DNA ends [51]–[54]. Under high oxygen tension, *Ogg1^−/−^* mouse cells displayed telomere attrition (Figure 2 and Figure 3). It is unclear if oxidative stress-induced DNA strand breaks can accumulate in telomeres due to unrepaired oxidative base lesions and contribute to telomere attrition in *Ogg1^−/−^* mouse cells. We thus examined the frequencies of genomic and telomeric DNA strand breaks in wild type and *Ogg1^−/−^* primary MEFs cultivated in 20% O_2_. γH2AX and XRCC1 are known to form foci at the sites of double strand breaks (DSBs) and SSBs, respectively [55]–[56], and formation of γH2AX and XRCC1 foci were therefore used as markers for DSBs and SSBs in the genome and telomeres.

**Figure 5:**
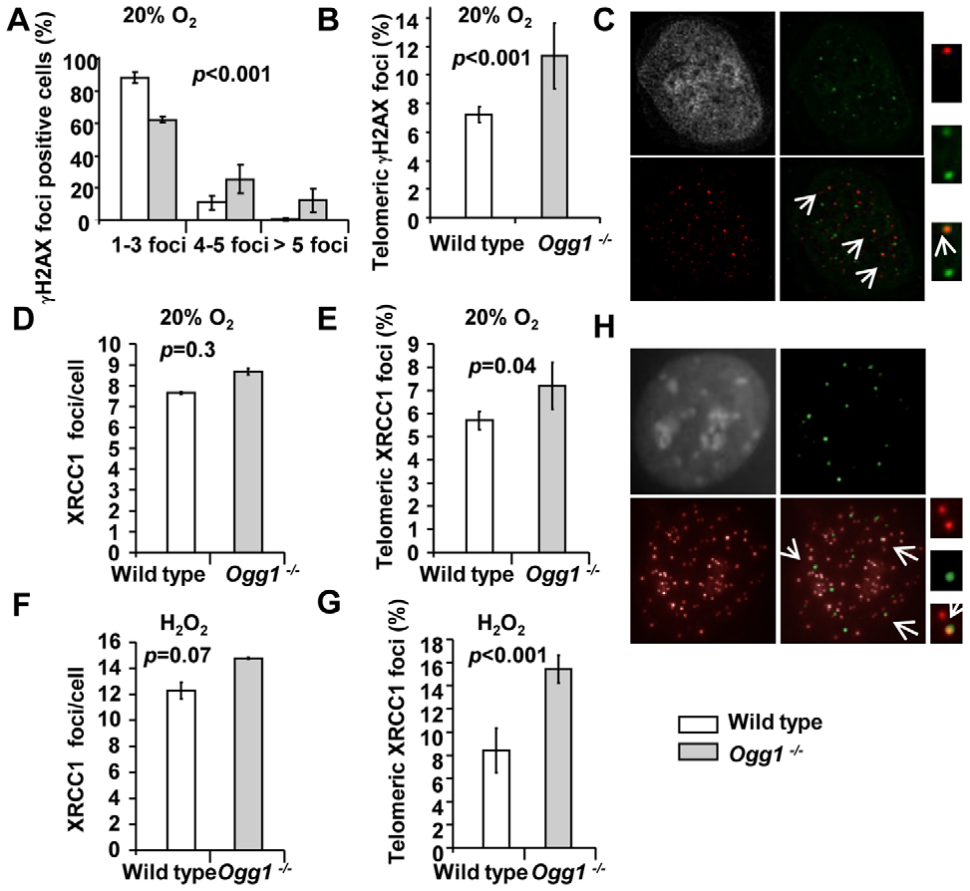
DNA damage foci in wild-type and *Ogg1^−/−^* primary MEFs cultivated in high oxidative environment. (A–C) γH2AX in late passage primary MEFs cultivated in 20% O_2_. (A) Percentage of γH2AX positive cells was divided into subgroups according to the number of foci in a cell. (B) Percentage of γH2AX foci in telomeres. (C) A representative *Ogg1^−/−^* primary MEF, showing DAPI staining (grey), γH2AX foci (green), and telomere fluorescence signals (red). Colocalization of γH2AX staining with telomere signal was illustrated in enlarged images at far right. (D–H) Genomic or telomeric XRCC1 foci in primary MEFs. (D,E) MEFs cultivated in 20% O_2_. (F,G) MEFs exposed to 10 µM H_2_O_2_ for 24 hours. Average genomic XRCC1 foci/cell (D or F) and percentage of XRCC1 foci in telomeres (E or G) were indicated. (H) A representative *Ogg1^−/−^* primary MEF cultivated in 20% O_2_, showing DAPI staining (grey), XRCC1 foci (green), and telomere fluorescence signals (red). Colocalization of XRCC1 signals with telomere signals was illustrated in enlarged images at far right.

γH2AX foci were detected in late passage wild type and *Ogg1^−/−^* MEFs by indirect immunofluorescence. A greater fraction of *Ogg1^−/−^* MEFs showed >3 γH2AX foci compared to the wild type (approximately 12% wild type and 38% *Ogg1^−/−^* MEFs, respectively) (Figure 5A). γH2AX foci were detected in telomeres in both wild type and *Ogg1^−/−^* MEFs by TEL-FISH, and the latter had approximately 2-fold more telomeric γH2AX foci (Figure 5B and 5C). To further clarify telomeric γH2AX foci, we examined the formation of 53BP1 foci in telomeres by TEL-FISH [57]. The 53BP1 foci were also detected in telomeric DNA in *Ogg1^−/−^* MEFs (Figure S5). Similarly, XRCC1 foci were found in the genome in both wild type and *Ogg1^−/−^* MEFs (Figure 5D), and *Ogg1^−/−^* MEFs had higher occurrence of telomeric XRCC1 foci (Figure 5E and 5H). This phenotype was further enhanced when MEFs were treated with 10 µM hydrogen peroxide for 24 hours (Figure 5F and 5G). Under low oxygen tension (i.e. 3% O_2_), the frequencies of γH2AX and XRCC1 foci were low, and no detectable difference was observed between wild type and *Ogg1^−/−^* MEFs (data not shown). Collectively, these results support the notion that oxidative stress can increase DSBs and SSBs in the genome and telomeres when *Ogg1* is deleted.

### High oxygen tension leads to preferential telomere strand loss in *Ogg1*-deficient primary MEFs

CO-FISH has been applied to detect defects in telomere lagging and leading strand loss, and it is proposed that such loss is caused by defects in lagging or leading strand synthesis (Figure 4A and 4B, and [58]–[59]). Because oxidative stress can increase DNA strand breaks in *Ogg1^−/−^* MEFs, it is possible that these DNA strand breaks may block telomere DNA replication and contribute to telomere attrition in *Ogg1^−/−^* MEFs. We therefore examined the frequencies of telomere lagging and leading strand loss in wild type and *Ogg1^−/−^* primary MEFs by CO-FISH. No significant difference in leading and/or lagging strand loss was detected between wild type and *Ogg1^−/−^* MEFs under low oxygen tension (3% O_2_); however, under high oxygen tension (20% O_2_), more telomere loss was found in the lagging strand in *Ogg1^−/−^* MEFs (1.80±0.37% and 3.50±0.44% lagging strand losses/chromosome in wild type and *Ogg1^−/−^* MEFs, respectively, *p*<0.001) (Figure 4D and Figure S6). These results indicate that oxidative stress-induced oxidative DNA lesions (possibly DNA strand breaks with adjacent oxidized guanines) may preferentially affect lagging strand DNA synthesis in telomeres in *Ogg1^−/−^* MEFs.

**Figure 6:**
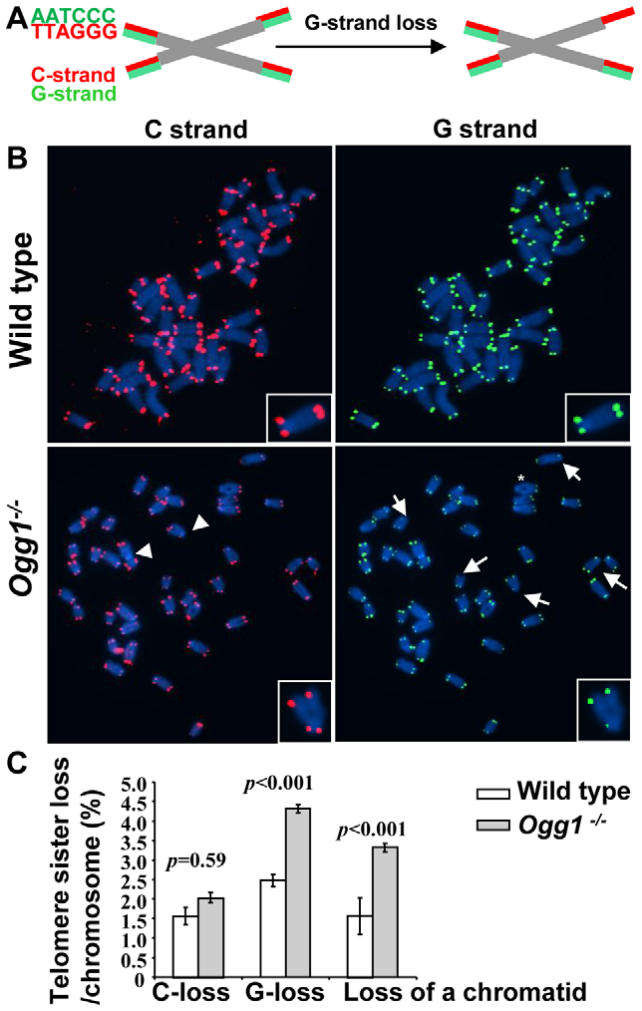
Preferential telomere loss in G strands in primary *Ogg1^−/−^* MEFs cultivated in 20% oxygen. (A) A schematic presentation of a two-color Q-FISH measurement of telomere signals on G- and C-strands by TRAMA-labeled (TTAGGG)_3_ PNA probe (red color) and FITC-labeled telomere (CCCTAA)_3 PNA_ probe (green color). (B) Representative metaphase spreads of wild-type and *Ogg1^−/−^* primary MEFs, showing DAPI staining (blue), C-strand (red), and G- strand (green) telomere fluorescence signals. (C) Percentage of telomere losses/chromosome in MEFs. Arrows: chromosomes with loss or reduced telomere signal intensity in G-strand. Arrowheads: chromosomes with loss or reduced telomere signal intensity in C-strand. *: chromosomes with loss of telomere signals in both G- and C-strands in a chromatid.

Aside from telomere lagging or leading strand synthesis defect, other factors (e.g. DNA strand breaks and nucleolytic degradation in a telomere strand) may also contribute to the loss of telomeric repeats in a telomere strand. To distinguish these possibilities, we employed a two-color telomere-FISH that detects telomere signals in G and C strands of a chromatid (Figure 6A and 6B). In 20% O_2_, loss of telomere signals in both G and C strands of a chromatid (or loss of a chromatid) was detected in wild type and *Ogg1^−/−^* MEFs, with higher frequencies in the latter (1.57±0.47% and 3.32±0.11% telomere chromatid losses/chromosome in wild type and *Ogg1^−/−^* MEFs, respectively, *p*<0.001) (Figure 6B and 6C). However, loss of telomere signal intensity in one of the telomere strands, either G or C strand was also evident in wild type and *Ogg1^−/−^* MEFs (Figure 6B and 6C), but G-strand loss appeared to be more prominent and was approximately 2-fold higher in *Ogg1^−/−^* MEFs (Figure 6C). Thus, telomere loss occurred in either one strand or both strands of a chromatid in *Ogg1^−/−^* MEFs. These results suggest that besides telomere replication defects, DNA breakage/degradation-mediated strand loss may have occurred in telomeres in *Ogg1^−/−^* MEFs. Because only the G-rich strand of telomeric DNA can harbor oxidized guanines, this may explain why oxidative DNA damage induces telomere attrition with strand bias.

### Deletion of mouse *Ogg1* is associated with increased oxidative guanine lesions in telomeres *in vivo*

**Figure 7:**
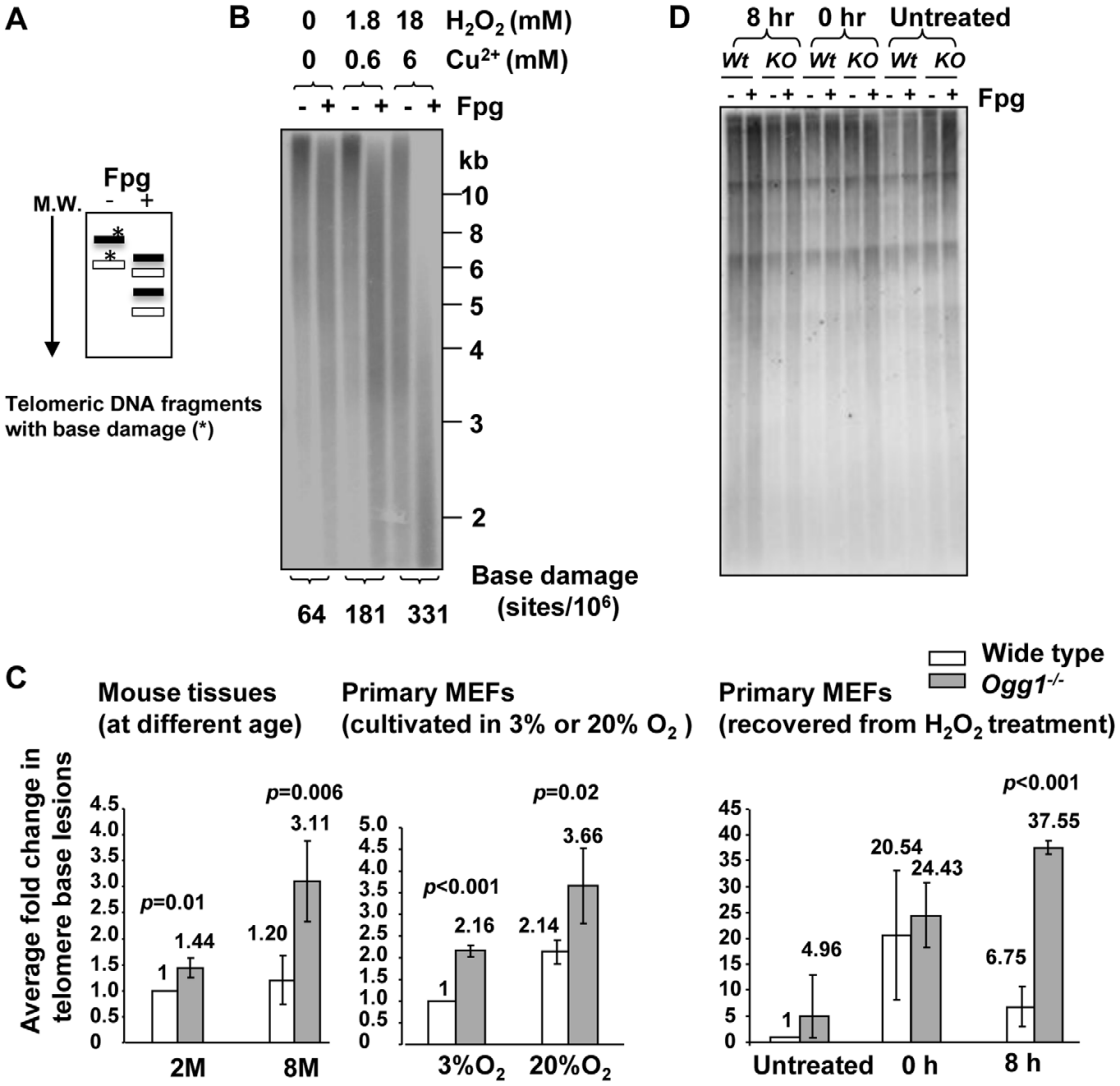
Increased level of Fpg-sensitive DNA lesions in telomeres in *Ogg1*-deficient mouse tissues and cells. (A) Schematics of telomere guanine damage detection. A telomere DNA fragment with guanine lesions is converted into smaller fragments due to Fpg's treatment. M.W: molecular marker from high (top) to low (bottom) molecular weight. (B) Validation of the method. Mouse genomic DNA was treated *in vitro* with increasing concentration of hydrogen peroxide (H_2_O_2_) plus Cu^2+^, followed by restriction enzyme and Fpg treatment. Telomeric DNA fragments with (+) or without (−) Fpg treatment were detected by Southern blot analysis using a radio-labeled telomere probe. Higher doses of H_2_O_2_ treatment caused Fpg-dependent increase of shorter telomere DNA fragments and more detectable Fpg-sensitive sites in the telomeric DNA. (C) Detection of telomeric base lesions in wild type and *Ogg1^−/−^* mouse tissues and primary MEFs. DNA was extracted from wild type and *Ogg1^−/−^* liver tissues from 2 and 8 month old mice (left panel), late passage primary MEFs cultured in 3% and 20% O_2_ (middle panel), or early passage primary MEFs collected at 0 hour and 8 hours after exposure to 500 µM H_2_O_2_ treatment for 60 minutes (right penal). In each experiment, fold changes in telomere base lesions in a testing sample were derived by normalizing the number of Fpg-sensitive lesions in the testing sample to that in a wild-type control. The control value was set to 1. The fold change represented average value from at least three independent experiments. M: month. H: hours. (D) Representative image of primary MEFs recovered from H_2_O_2_ treatment. 8 hours after exposure to H_2_O_2_, *Ogg1^−/−^* MEFs still showed smaller telomere DNA fragments after Fpg treatment, while wild type MEFs displayed similar distribution of telomere DNA fragments with or without Fpg treatment.

Guanine has a lower oxidation potential compared to other bases, and triple guanines, composed of the mammalian telomere repeats, have an even lower oxidation potential [60]–[61]. Consistently, it has been found that triple guanines in telomere repeats are prone to oxidative damage *in vitro* [23]–[25], [62]. To determine the level of guanine oxidation in telomeres *in vivo*, genomic DNAs from wild-type and *Ogg1* deficient mouse liver and primary MEFs were digested with restriction enzymes and then examined for their sensitivity to *E. coli* Fapy DNA glycosylase (Fpg). Fpg excises oxidized guanines, resulting in abasic sites that are further processed by the lyase activity of Fpg to create SSBs [27]–[28]. The extent of increased smaller single stranded telomeric DNA fragments is proportional to the amount of Fpg-sensitive lesions present within the telomeric DNA and can be extrapolated to estimate the number of lesions (Figure 7A and Figure S7, and [48]).

To validate the method, genomic DNA was treated *in vitro* with increasing concentration of hydrogen peroxide (H_2_O_2_) plus Cu^2+^. Higher doses of H_2_O_2_ treatment caused detectable increase of Fpg-sensitive lesions (Figure 7B), demonstrating that the method is feasible in estimating Fpg-sensitive lesions in telomeres. Next, we measured Fpg-sensitive lesions in telomeres in mouse livers derived from wild type and *Ogg1^−/−^* mice. The level of telomeric Fpg-sensitive lesions was not significantly changed in the hepatocytes from 2- and 8-month old wild type mice; however, telomeric Fpg-sensitive lesions were elevated in the hepatocytes from 8-month old *Ogg1^−/−^* mice (Figure 7C). We also measured oxidative guanine lesions in primary MEFs during prolonged culture under low and high oxygen tensions (3% O_2_ and 20% O_2_). Higher levels of Fpg-sensitive lesions were observed in primary MEFs under high oxygen tension, and *Ogg1^−/−^* MEFs harbored more lesions than wild type MEFs (Figure 7C). Collectively, these results indicate that ablation of OGG1 function can increase oxidative guanine lesions in telomeres in mouse tissues with aging or in primary MEFs during prolonged culture or under oxidative stress conditions.

To further verify if OGG1 participates in oxidative guanine repair in telomeres *in vivo*, early passage wild type and *Ogg1^−/−^* primary MEFs were exposed to 500 µM hydrogen peroxide for 60 minutes and then allowed to recover for 8 hours. Immediately after hydrogen peroxide treatment (0 hour), high levels of Fpg-sensitive lesions were detected in wild type and *Ogg1^−/−^* MEFs, compared to untreated MEFs. Eight hours after removal of hydrogen peroxide, Fpg-sensitive lesions were significantly reduced in wild type MEFs; in contrast they remained at a higher level in *Ogg1^−/−^* MEFs (Figure 7C and 7D). Thus, *Ogg1^−/−^* MEFs were inefficient in the repair of hydrogen peroxide-induced Fpg-sensitive lesions in telomeres, while wild type MEFs repaired these lesions with high efficiency. These results demonstrate that OGG1 is involved in the repair of oxidative guanine lesions in telomeres *in vivo*.

## Discussion

BER is the primary DNA repair pathway for the repair of oxidative base lesions. Here, we studied the impact of *Ogg1* deficiency on telomeres in mammalian cells. We found that ablation of OGG1 function resulted in increased oxidative guanine lesions in telomeres in mice with aging or in primary MEFs during prolonged culture or cultivated in a high oxidative environment. In addition, lack of *Ogg1* led to telomere length alteration that was dependent on the level of oxidative stress. Furthermore, deletion of *Ogg1* caused altered recombination, increased DNA strand breaks, and preferential strand loss in telomeres. Our data support that oxidative guanine lesions affect telomere integrity and that the OGG1-initiated BER pathway plays an important role in telomere base damage repair and telomere maintenance in mammals.

*Ogg1* deficient mouse cells showed moderate telomere lengthening under low oxygen tension (e.g. in tissues or 3% O_2_); however, they displayed accelerated telomere shortening under high oxygen tension (20% O_2_) or with paraquat treatment. This observation suggests that the level and types of oxidative DNA damage in telomeres may affect the outcome of telomere length. Several possibilities may contribute to the telomere length alteration in *Ogg1* deficient mouse cells.

8-oxoG can directly disrupt telomeric DNA binding by TRF1 and TRF2 [26], and unrepaired 8-oxoG can lead to GC to TA transversions [33]. The affinity of telomere binding proteins to telomeric DNA is sequence-specific and can be altered by mutations in telomeric DNA [63]. Thus, both base lesions and base lesion-induced mutations may affect the association of telomere binding proteins to telomeres. Opresko et al have previously shown that the level of 8-oxoG in telomeres adversely affects binding by telomere binding proteins [26]. Thus, the number of oxidative base lesions and mutations may determine the severity of telomere binding protein depletion in telomeres. It is known that reduced binding or severe loss of telomere binding proteins in telomeres can lead to different telomere phenotypes; the former causes telomere lengthening and the latter results in telomere uncapping [3]. Thus, when few base lesions affect telomeric DNA repeats, they may moderately reduce telomere binding proteins in telomeres, which could liberate the negative regulation of telomere binding proteins on telomerase and consequently increase telomerase-dependent telomere repeat additions. Our studies in *S. cerevisiae* support this notion, in which telomere lengthening in *ogg1*-deleted *S. cerevisiae* is dependent on telomerase-mediated telomere elongation [50]. On the other hand, once oxidized bases accumulate to a certain level in telomeres, they may severely deplete telomere binding proteins in telomeres and result in telomere uncapping. Uncapped telomeres can become targets for nucleolytic degradation and hence cause telomere shortening.

When exposed to 20% O_2_ or hydrogen peroxide, *Ogg1* deficient mouse cells showed increased incidences of SSBs and DSBs in telomeres, evident by XRCC1 and γH2AX foci formation in telomeres. These DNA strand breaks can represent an obstacle for DNA replication. Furthermore, base lesions in the vicinity of DNA strand breaks can somehow accelerate end resection, possibly by stimulating an endonuclease activity close to the breaks [64]. As a result, these DNA defects may ultimately lead to telomere shortening in *Ogg1* deficient mouse cells. However, fewer DNA strand breaks may also arise in *Ogg1* deficient mouse tissues and partially inhibit DNA replication, which could consequently enhance telomerase pathway and induce telomere elongation [65]–[66]. DNA strand breaks in *Ogg1* deficient mouse cells may arise in telomeres by several means. High oxygen tension has been shown to cause detectable levels of SSBs in telomeres, especially in the G-strand [22]. Since the presence of oxidative guanine damage in the vicinity of DNA breakages may impose a hindrance to the resolution of DNA ends [51]–[54], they may possibly inhibit repair of DNA strand breaks in telomeres. If adenine is incorporated opposite unrepaired 8-oxoG, removal of adenine by the MYH DNA glycosylase and subsequent abasic site processing can lead to SSBs in the C-strand [40]. SSBs may also be indicative of increased partial repair products of back-up DNA glycosylase activity, e.g. Neil1 [67].

Previous reports demonstrate that telomere lagging strand loss can be detected in WRN and FEN1 mutant cells via CO-FISH, which may reflect lagging strand telomere synthesis defects [58]–[59]. Because oxidative guanine lesions are located at G-strand in telomeres, they may preferentially inhibit repair of SSBs in this strand. As a result, lagging strand telomere synthesis may be affected. Indeed, *Ogg1* deficient MEFs showed an increase in telomere lagging strand loss by CO-FISH analysis. This result suggests that oxidative guanine damage and/or its negative effect on the repair of telomere strand breaks may perturb lagging strand DNA synthesis in telomeres. Telomere lagging strand loss may also result from SSBs in G-strand along with loss of distal telomeres or nucleolytic degradation. In fact, the two-color Q-FISH showed that telomeric G-strand loss can occur alone without a loss of its complementary C-strand in *Ogg1^−/−^* MEFs, supporting the latter possibility.

Besides changes in telomere length, the incidence of T-SCEs either increased or decreased in *Ogg1* deficient mouse cells, which was reversely associated with the level of oxidative stress. Several possibilities may account for the altered telomere recombination in *Ogg1* deficient mouse cells. First, variable levels of oxidative base damage may have different impact on recombination activity. For example, recombination rates are substantially increased in BER deficient yeast cells harboring low levels of oxidative DNA damage in the genome, and it has been postulated that a moderately damaged genome could promote illegitimate recombination that serves as a compensatory response in order to tolerate oxidative DNA damage [68]. However, high density of oxidative base lesions can inhibit RAD52 annealing activity and thus result in reduced recombination resolution [51]. Second, OGG1 can inhibit RAD52 strand annealing and exchange activity [51], and removal of OGG1 would therefore relieve this inhibition and activate the recombination pathway. Third, oxidized guanines may affect telomere recombination by disrupting shelterin's association to telomeres. Previous studies demonstrate that telomere binding proteins prevent telomeres from becoming substrates for HR, and deletion of telomere binding proteins invokes T-SCE events [10], [15]. Because oxidative base lesions in telomeric DNA can attenuate binding by telomere binding proteins [26], it is possible that reduced binding of telomere binding proteins to telomeres may promote telomere recombination. Finally, increased telomere sister chromatid exchanges may associate with recombination repair of stalled or broken replication forks that might occur at the sites of oxidative bases and/or DNA strand breaks in telomeres.

We found that oxidative guanine lesions in telomeres were elevated in older animals or in primary MEFs cultivated under oxidative stress conditions. Thus, oxidative damage on guanine bases can increase in telomeres in aging or by environmental oxidative stress. The level of Fpg-sensitive lesions in telomeres was increased in *Ogg1^−/−^* mouse liver and primary MEFs, in comparison to their wild type counterparts. These results indicate that OGG1 is involved in repairing oxidative guanine lesions in telomeres *in vivo*. This view was further supported by the evidence that *Ogg1^−/−^* MEFs were defective in the repair of hydrogen peroxide-induced Fpg-sensitive lesions in telomeres.

**Figure 8:**
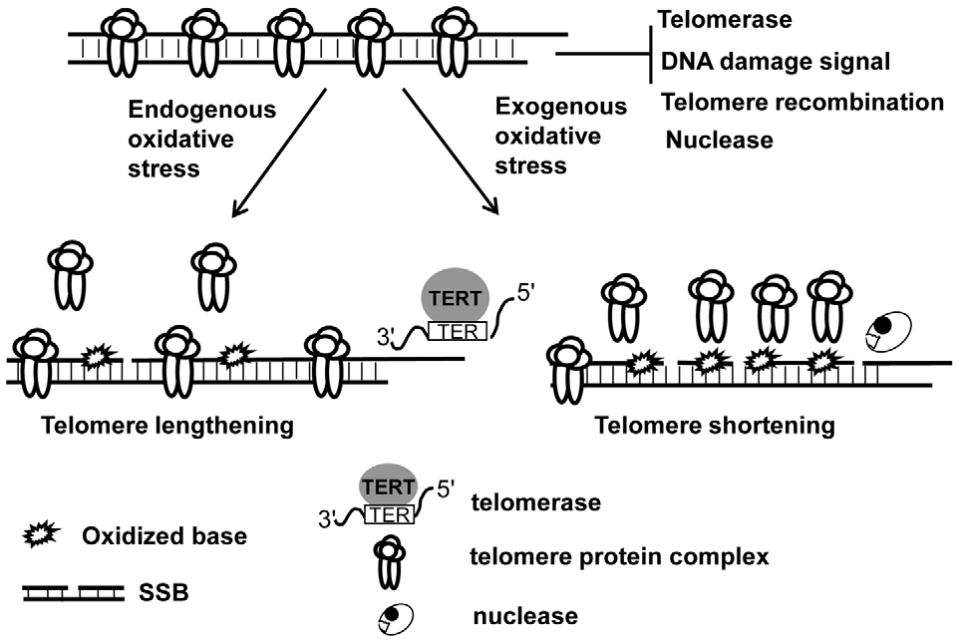
The levels and types of oxidative DNA damage may determine telomere length alteration. Telomeres are normally capped by telomere protein complex which limit the access of telomerase to telomeres and prevent telomeres from evoking DNA damage response and becoming the subject of nucleolytic degradation and recombination. Under low oxidative condition, base damage is low and affects fewer telomeric DNA repeats, which may moderately reduce telomere protein complex in telomeres, thus causing increased telomerase-mediated telomere lengthening. High oxidative stress can, however, not only increase base damage but also induce DNA strand breaks, and the former may severely deplete telomere protein complex in telomeres and impose a hindrance to the resolution of DNA breaks. As a result, it may lead to telomere uncapping, increased telomere strand breaks, and nucleolytic degradation, hence causing telomere shortening.

Oxidative stress-induced SSBs can cause telomere shortening in mammalian cells [20]. Here, we report that another form of oxidative DNA damage, oxidative base lesions can induce either telomere lengthening or shortening, depending on the level of oxidative stress. In a given organism, telomere length is maintained via a balance between telomere elongation and shortening [1]. It is possible that moderate oxidative base damage may favor the pathways for telomere lengthening (e.g. telomerase), while extensive oxidative base damage may attenuate telomere capping, telomere recombination, telomere replication, and the resolution of DNA strand breaks and ultimately result in telomere attrition (Figure 8). Telomere shortening has been linked to human aging and cancer development. Perhaps extensive base damage occurs in individuals with the conditions, such as defective BER and increased ROS levels (for example, chronic inflammation), which may consequently lead to accelerated telomere attrition, thus contributing to premature aging and cancer formation.

## Supporting Information

Figure S1 Q-FISH analysis of mouse bone marrow cells from 12-month-old wild type and *Ogg1^−/−^* mice. (A) Representative metaphase spreads of wild type and *Ogg1^−/−^* mouse bone marrow cells showing DAPI staining (blue, upper panel) and telomere fluorescence signals (red, upper panel; white, lower panel). Quantitative measurement and dynamic range of telomeric DNA signal intensity at individual chromosome ends are shown as histogram (B) and box-plot (C). An increase in telomere signal intensity was observed in *Ogg1^−/−^* mice.
(0.90 MB TIF)

Figure S2 Q-FISH analysis of telomere length in activated mouse splenocytes cultivated in 20% O_2_. (A) Representative metaphase spreads of wild type and *Ogg1^−/−^* mouse splenocytes. Quantitative measurement and dynamic range of telomeric DNA signal intensity at individual chromosome ends are shown as histogram (B) and box-plot (C). A decrease in telomere signal intensity was observed in mouse *Ogg1^−/−^* splenocytes. Arrows: chromosome ends without detectable telomere signals.
(0.47 MB TIF)

Figure S3 Q-FISH analysis of telomere length in mouse bone marrow cells subcultured with paraquat. (A) Representative metaphase spreads of wild type and *Ogg1^−/−^* mouse bone marrow cells. Quantitative measurement and dynamic range of telomeric DNA signal intensity at individual chromosome ends are shown as histogram (B) and box-plot (C). A decrease in telomere signal intensity was observed in mouse *Ogg1^−/−^* bone marrow cells.
(0.52 MB TIF)

Figure S4 Telomerase activity in wild type and *Ogg1^−/−^* mouse bone marrow cells. qT-PCR analysis was performed on bone marrow cell lysate at indicated concentration. The Ct value was converted into log value. A comparable telomerase activity was detected in wild type and *Ogg1^−/−^* mouse bone marrow cells.
(0.15 MB TIF)

Figure S5 53BP1 foci are detected in telomeres in *Ogg1^−/−^* MEFs. Upper panel: a representative *Ogg1^−/−^* late passage primary MEF, showing DAPI staining (blue), 53BP1 foci (green), and telomere fluorescence signals (red). Arrows: colocalization of 53BP1 staining with telomere signal. Lower panel: a primary MEF negative for 53BP1 foci.
(0.42 MB TIF)

Figure S6 CO-FISH analysis of primary *Ogg1^−/−^* MEFs. Individual images represent leading-strand (red) and lagging-strand (green) telomere fluorescence signals. Chromosomes without telomere loss had two telomere fluorescence signals in each image. Merged images were shown at the bottom.
(0.32 MB TIF)

Figure S7 Schematics of telomerase base lesion calculation. (A) Gel profiles of wild type and *Ogg1^−/−^* mouse cells with or without Fpg treatment. (B) The density in each data point was measured by densitometer and ImageQuant software and collected into a grid. (C) The histogram illustrates a density profile of a grid and the corresponding molecular size at each data point. The mean length (ML) was calculated as a center of mass, and the frequencies of Fpg-sensitive lesions in a sample were based on ML values, as described in Methods.
(0.40 MB TIF)
