# Supplementary figures and images for: Characterization of Oxidative Guanine Damage and Repair in Mammalian Telomeres
Source: PLoS Genet. 2010 May 13;6(5):e1000951. doi: 10.1371/journal.pgen.1000951 (PMC2869316; doi:10.1371/journal.pgen.1000951)

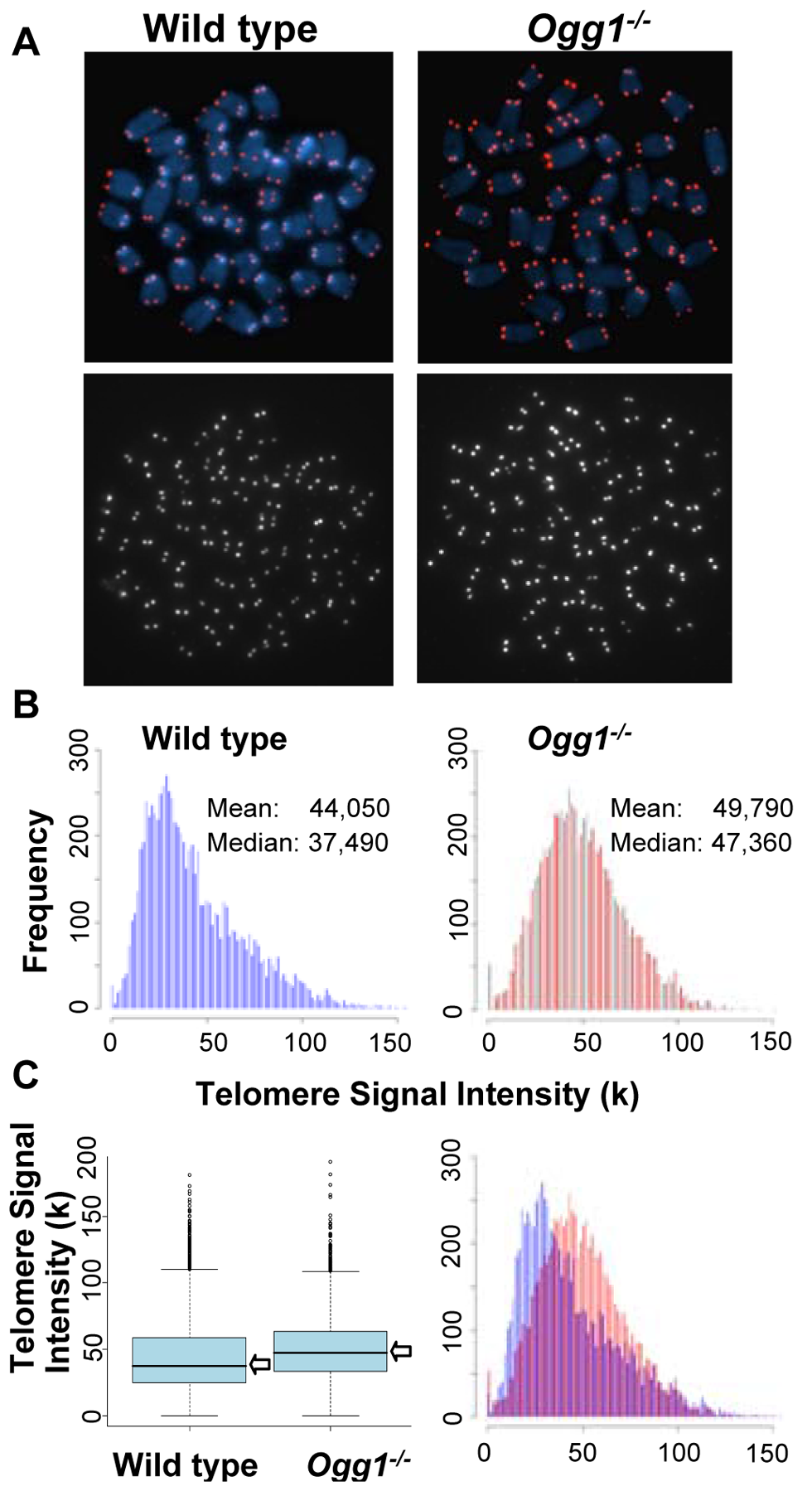

Supplement: Figure S1 — Q-FISH analysis of mouse bone marrow cells from 12-month-old wild type and Ogg1−/− mice. (A) Representative metaphase spreads of wild type and Ogg1−/− mouse bone marrow cells showing DAPI staining (blue, upper panel) and telomere fluorescence signals (red, upper panel; white, lower panel). Quantitative measurement and dynamic range of telomeric DNA signal intensity at individual chromosome ends are shown as histogram (B) and box-plot (C). An increase in telomere signal intensity was observed in Ogg1−/− mice. (0.90 MB TIF) [file pgen.1000951.s001.tif]

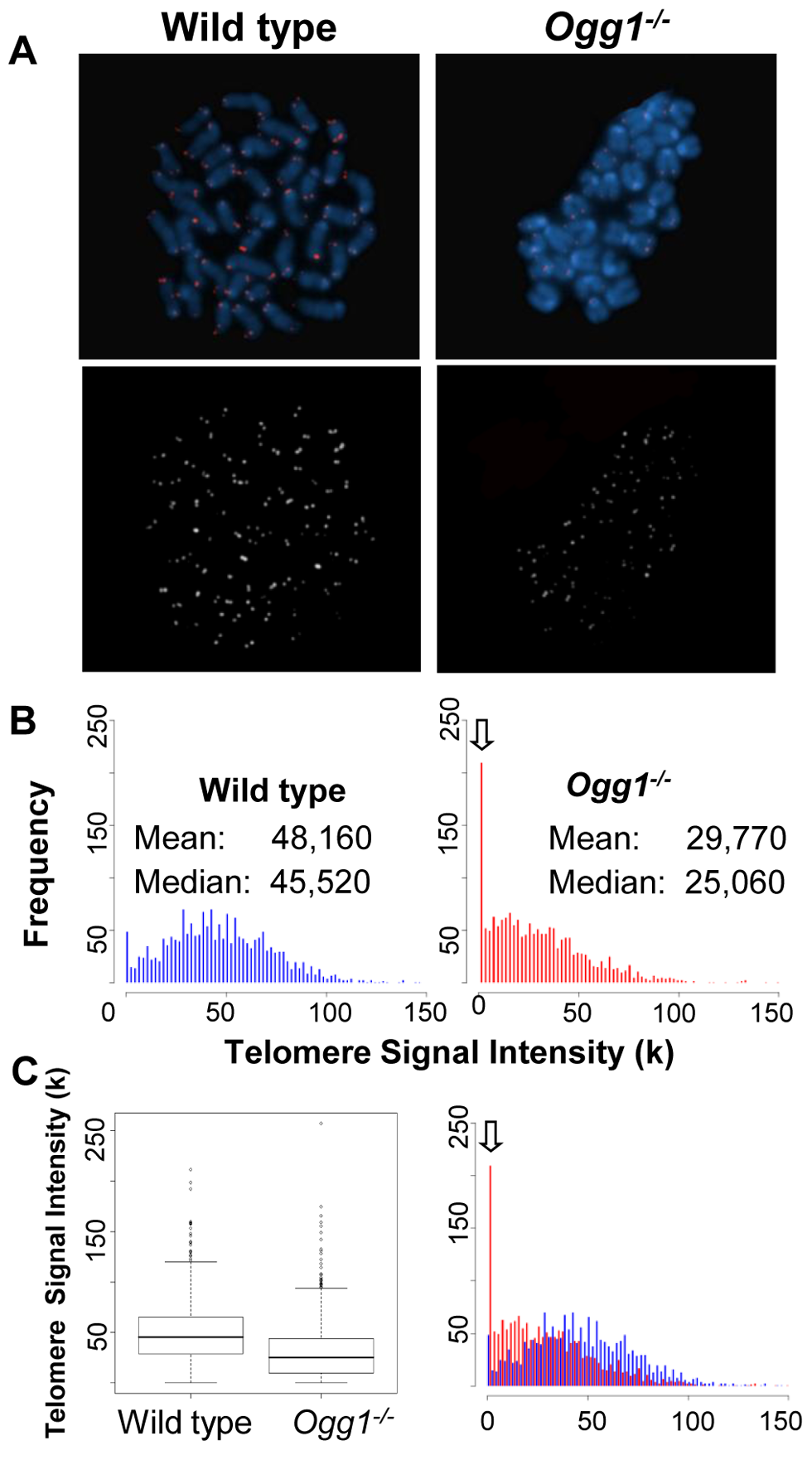

Supplement: Figure S2 — Q-FISH analysis of telomere length in activated mouse splenocytes cultivated in 20% O2. (A) Representative metaphase spreads of wild type and Ogg1−/− mouse splenocytes. Quantitative measurement and dynamic range of telomeric DNA signal intensity at individual chromosome ends are shown as histogram (B) and box-plot (C). A decrease in telomere signal intensity was observed in mouse Ogg1−/− splenocytes. Arrows: chromosome ends without detectable telomere signals. (0.47 MB TIF) [file pgen.1000951.s002.tif]

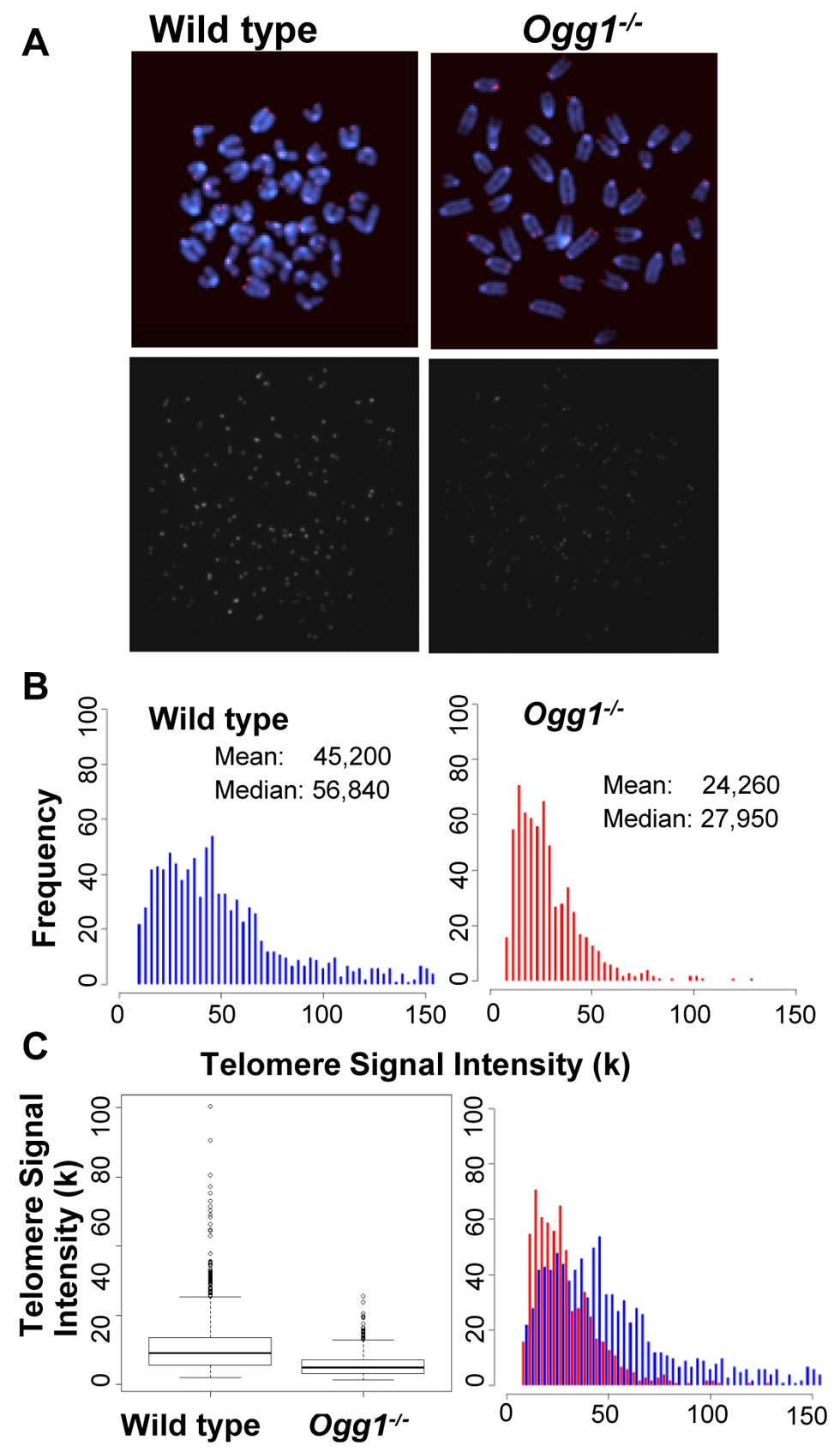

Supplement: Figure S3 — Q-FISH analysis of telomere length in mouse bone marrow cells subcultured with paraquat. (A) Representative metaphase spreads of wild type and Ogg1−/− mouse bone marrow cells. Quantitative measurement and dynamic range of telomeric DNA signal intensity at individual chromosome ends are shown as histogram (B) and box-plot (C). A decrease in telomere signal intensity was observed in mouse Ogg1−/− bone marrow cells. (0.52 MB TIF) [file pgen.1000951.s003.tif]

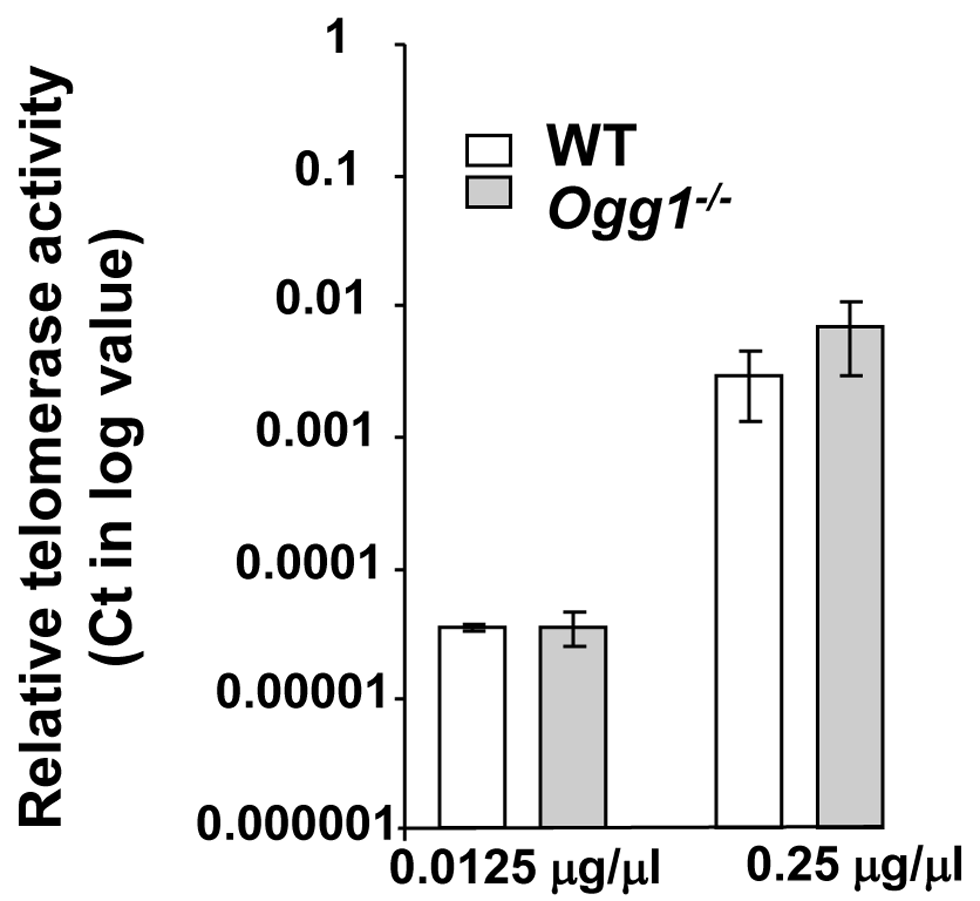

Supplement: Figure S4 — Telomerase activity in wild type and Ogg1−/− mouse bone marrow cells. qT-PCR analysis was performed on bone marrow cell lysate at indicated concentration. The Ct value was converted into log value. A comparable telomerase activity was detected in wild type and Ogg1−/− mouse bone marrow cells. (0.15 MB TIF) [file pgen.1000951.s004.tif]

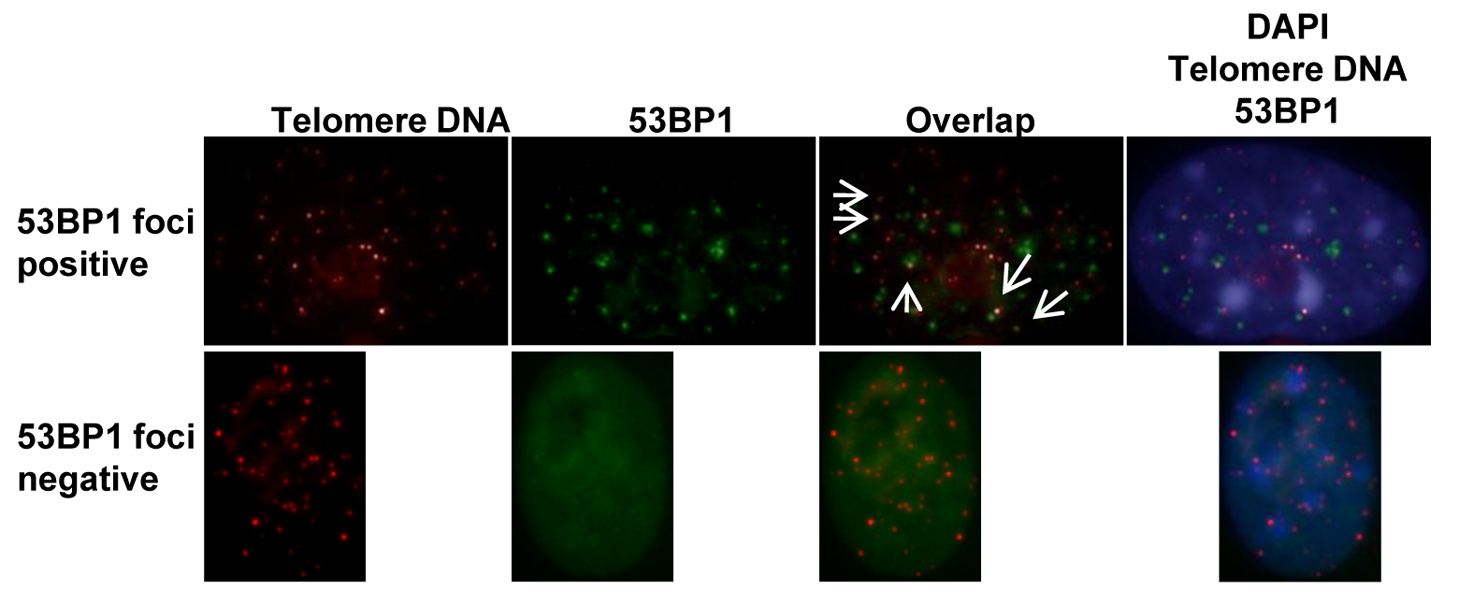

Supplement: Figure S5 — 53BP1 foci are detected in telomeres in Ogg1−/− MEFs. Upper panel: a representative Ogg1−/− late passage primary MEF, showing DAPI staining (blue), 53BP1 foci (green), and telomere fluorescence signals (red). Arrows: colocalization of 53BP1 staining with telomere signal. Lower panel: a primary MEF negative for 53BP1 foci. (0.42 MB TIF) [file pgen.1000951.s005.tif]

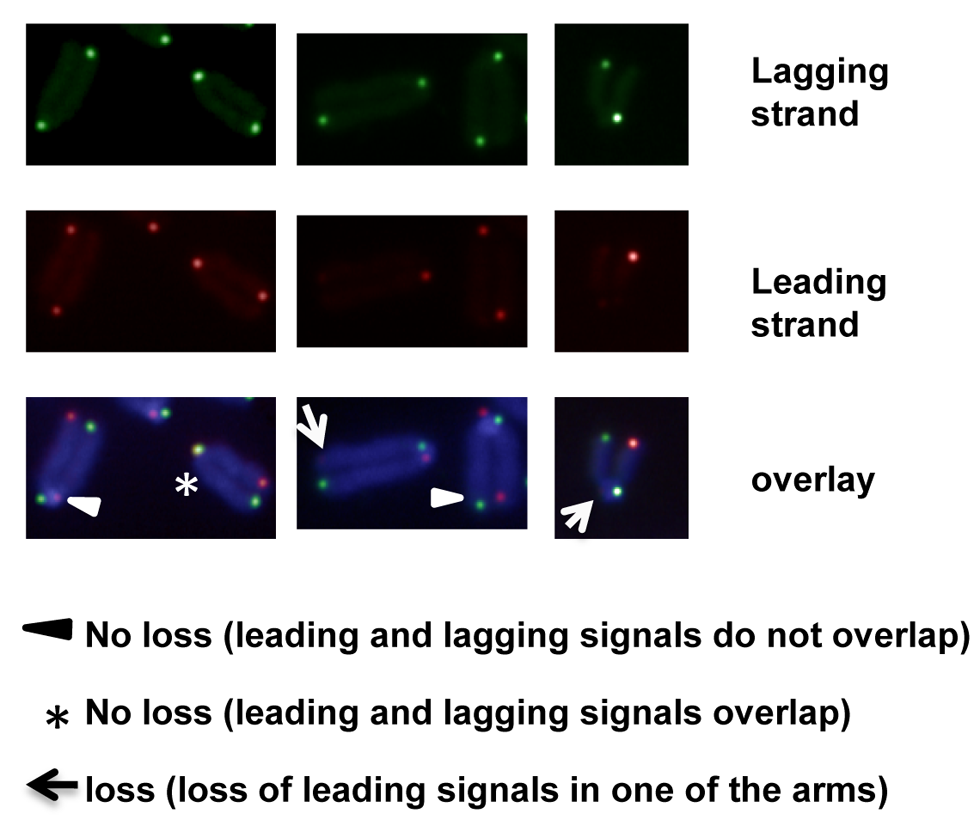

Supplement: Figure S6 — CO-FISH analysis of primary Ogg1−/− MEFs. Individual images represent leading-strand (red) and lagging-strand (green) telomere fluorescence signals. Chromosomes without telomere loss had two telomere fluorescence signals in each image. Merged images were shown at the bottom. (0.32 MB TIF) [file pgen.1000951.s006.tif]

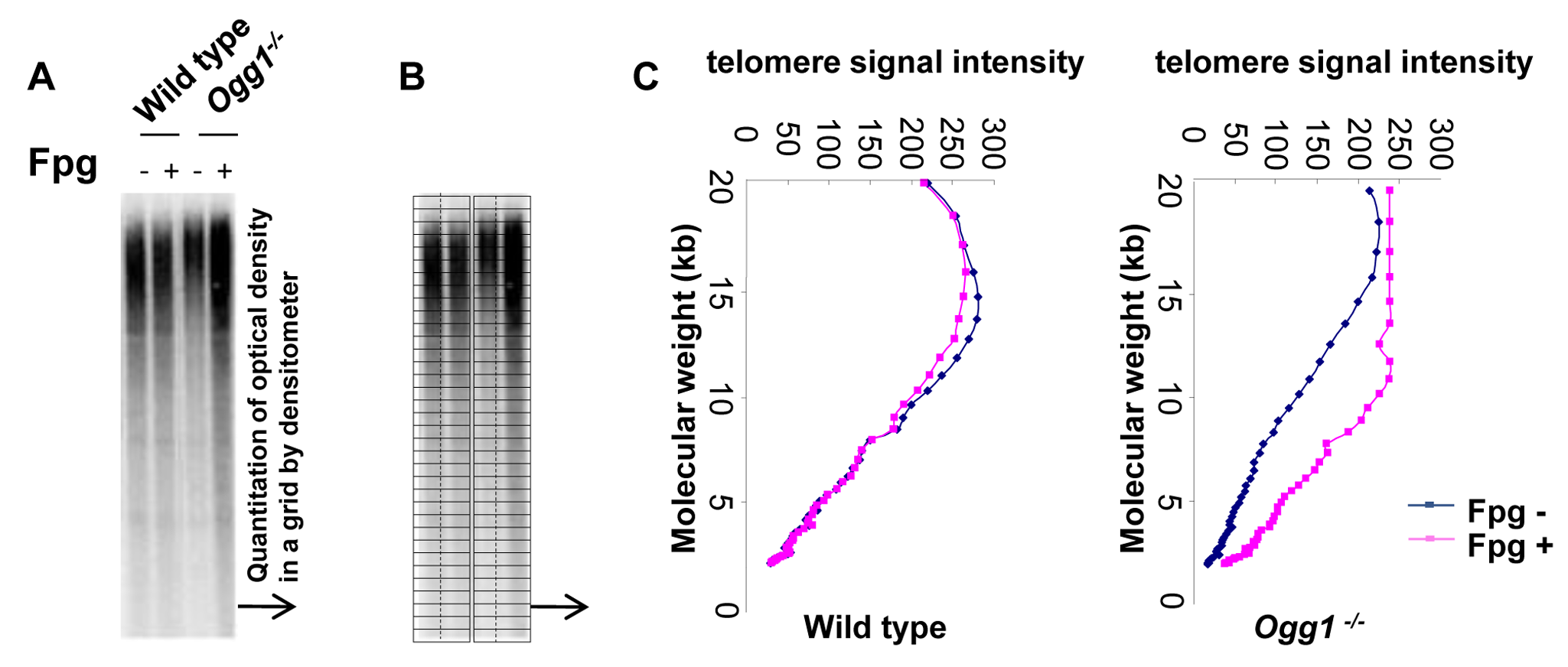

Supplement: Figure S7 — Schematics of telomerase base lesion calculation. (A) Gel profiles of wild type and Ogg1−/− mouse cells with or without Fpg treatment. (B) The density in each data point was measured by densitometer and ImageQuant software and collected into a grid. (C) The histogram illustrates a density profile of a grid and the corresponding molecular size at each data point. The mean length (ML) was calculated as a center of mass, and the frequencies of Fpg-sensitive lesions in a sample were based on ML values, as described in Methods. (0.40 MB TIF) [file pgen.1000951.s007.tif]
